# Hirayama disease: diagnostic essentials in neuroimaging

**DOI:** 10.1002/ccr3.1246

**Published:** 2017-10-30

**Authors:** Stylianos Kapetanakis, Danae Chourmouzi, Aikaterini Terzoudi, Nikiforos Georgiou, Eirini Giovannopoulou

**Affiliations:** ^1^ Spine Department and Deformities Interbalkan European Medical Center Thessaloniki Greece; ^2^ Department of Radiology Interbalkan European Medical Center Thessaloniki Greece

**Keywords:** Hirayama disease, neuroimaging, spine

## Abstract

A 22‐year‐old male presented with progressive muscular weakness of the upper extremities. MRI of the cervical spine established the final diagnosis of Hirayama disease (HD). HD is a rare disease with benign progress. Neurologists and radiologists should be aware of the specific neuroimaging signs of this rare clinical entity.

## Case Description

**Figure 1:**
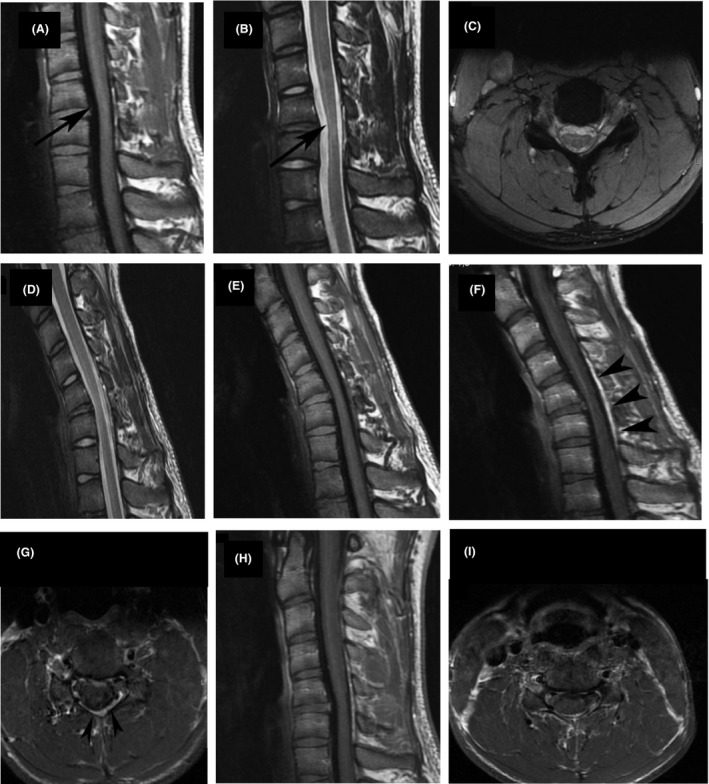
Neutral position MRI of the spine, sagittal T1‐weighted image (A) and T2‐weighted image (B) showing straightening of the cervical curvature and localized cord atrophy at C5‐C6 level (arrows).On axial T2‐weighted image (C) spinal cord shows normal signal intensity. Flexion sagittal T2‐weighted image (D) and T1‐weighted (E) showing anterior displacement of dorsal dura and a crescent epidural compartment which shows enhancement on postcontrast sagittal (F) and axial (G) T1‐weighted images (arrowheads). Repeated acquisition on neutral position shows elimination of enhancement on sagittal (H) and axial (I) postcontrast T1‐weighted images.

A 22 years old male patient presented with deteriorating bilateral muscular atrophy and tremor of the distal upper extremities with insidious onset 5 years ago. Cold exposure worsened the symptomatology. Clinical examination revealed symmetrical muscular atrophy of the hands and forearms and reduced muscular strength during flexion, extension, and abduction of the fingers and flexion, extension of the wrist, as well as reduced tendon reflexes from the affected neurotomes. Electroneurophysiology and electromyography showed signs of chronic motor neuron lesion at C7, C8, and T1 myelotomes, bilaterally. Magnetic resonance images (MRI) acquired in neutral position showed loss of cervical lordosis and focal cord atrophy at C5‐C6 level. MRI acquired in flexion revealed widening of the posterior epidural space. On postcontrast images, uniform enhancement of the epidural space was observed. The enhancement was reduced in the neutral position. According to the aforementioned findings described also in Figure 1, the diagnosis of HD was finally made and the patient was treated conservatively.

HD is a rare clinical entity, first described by Hirayama in 1959 1. However, neurologists and radiologists should be familiar with the imaging findings of the disease as MRI in neck flexion is the key for diagnosis 2.

## Authorship

KS: designed and drafted the manuscript, CD and TA: acquired all MRI images and other pertinent patient data, GN: contributed to the analysis of the existing data, GE: designed and supervised the study.

## Conflict of Interest

The authors declare that they have no competing interests.
